# Carbonylative synthesis and functionalization of indoles

**DOI:** 10.3762/bjoc.20.87

**Published:** 2024-04-30

**Authors:** Alex De Salvo, Raffaella Mancuso, Xiao-Feng Wu

**Affiliations:** 1 Laboratory of Industrial and Synthetic Organic Chemistry (LISOC), Department of Chemistry and Chemical Technologies, University of Calabria, Via Pietro Bucci 12/C, 87036 Arcavacata di Rende (CS), Italy; 2 Leibniz-Institut für Katalyse e.V., Albert-Einstein-Str. 29a, 18059 Rostock, Germany; 3 Dalian National Laboratory for Clean Energy, Dalian Institute of Chemical Physics, Chinese Academy of Sciences, 116023 Liaoning, China

**Keywords:** carbonylation, functionalization, indole, metal catalyst, organometallic chemistry

## Abstract

Carbonylation processes have become widely recognized as a versatile, convenient, and low-cost method for the synthesis of high-value compounds. Given the great importance of heterocyclic compounds, the carbonylative approach has become increasingly important for their synthesis. In this mini-review, as a class of benzo-fused nitrogen-containing heterocyclic compounds, we summarized and discussed the recent achievements on the synthesis and functionalization of indole derivatives via carbonylative approaches.

## Introduction

Indole is a heterocyclic compound consisting of a benzene ring fused with a pyrrole ring. It was discovered in 1866 by Baeyer and Knop as the basic structure of the natural dye indigo, from which it is derived [1]. The indole ring is a common structural element found in both natural and synthetic products, including pharmaceuticals, agrochemicals, dyes, herbicides, and materials [2–4]. The indole core is particularly noteworthy for its role in various biologically active compounds and drugs, such as antihypertensives, anti-inflammatories, antimycotics, antimigrants, anticancer drugs, and many others [5–7]. The first synthesis of indole has been introduced by Fischer in 1883 and involves its synthesis from phenylhydrazine and an aldehyde or ketone using an appropriate acid catalyst [8]. In the following years, new processes were developed for the synthesis of indole such as the Castro, Bischler, and Larock synthesis etc. [2,9–10]. Carbonylation reactions represent a powerful method for the introduction of a C1 building block into organic substrates using carbon monoxide, its surrogates, or compounds able to act as carbon monoxide sources [11]. In recent years, many groups have used the carbonylation approach for the synthesis and functionalization of indoles, which is what we are discussing in this mini-review.

## Review

### Carbonylative synthesis of indoles

#### Synthesis of indoles by Pd(0)-catalyzed carbonylation reaction of halide compounds

**Scheme 1:**
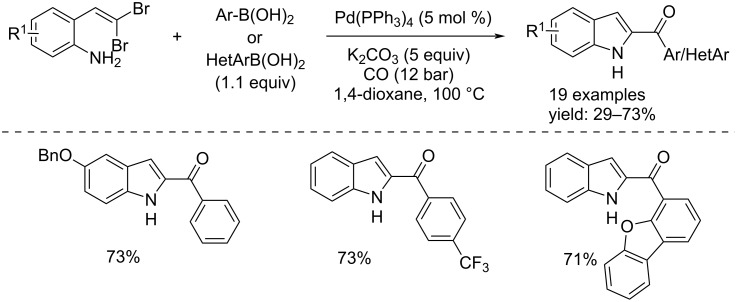
Pd(0)-catalyzed domino C,N-coupling/carbonylation/Suzuki coupling reaction for the synthesis of 2-aroylindoles and 2-heteroaroylindoles.

Processes using organic halides as their starting materials involving the oxidative addition of Pd(0) to C–X bonds to give Ar–Pd^II^–X complexes are important for the synthesis of heterocyclic and non-heterocyclic compounds. In 2009, Arthuis and co-workers developed a new process for the synthesis of 2-aroylindoles and 2-heteroaroylindoles by a one-pot palladium-catalyzed domino reaction that involves an initial C,N-coupling followed by carbon monoxide insertion, and Suzuki–Miyaura coupling reaction, from 2-*gem-*dibromovinylaniline [12]. In the presence of Pd(PPh_3_)_4_ (5 mol %) as catalyst, 5 equivalents of base (K_2_CO_3_), an aryl- or heteroarylboronic ester (1.1 equivalents), CO (12 bar), in dioxane at 100 °C after 16 h the indole derivatives were isolated with good yields (Scheme 1).

**Scheme 2:**
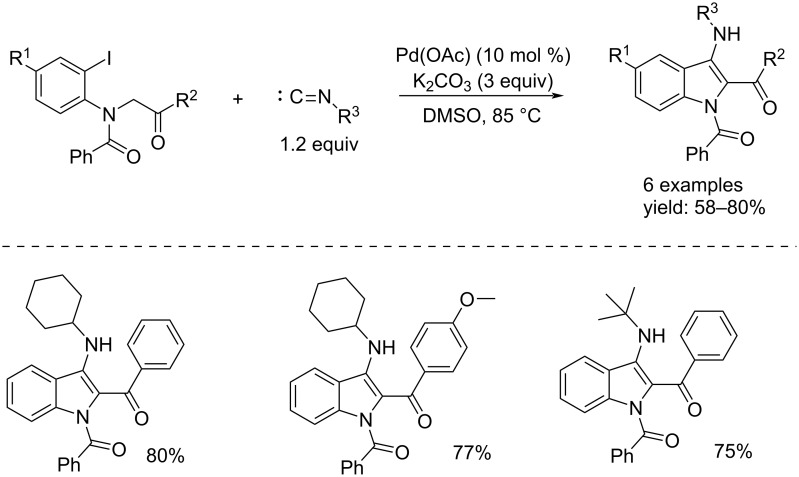
Pd(0)-catalyzed single isonitrile insertion: synthesis of 1-(3-amino)-1*H*-indol-2-yl)-1-ketones.

**Scheme 3:**
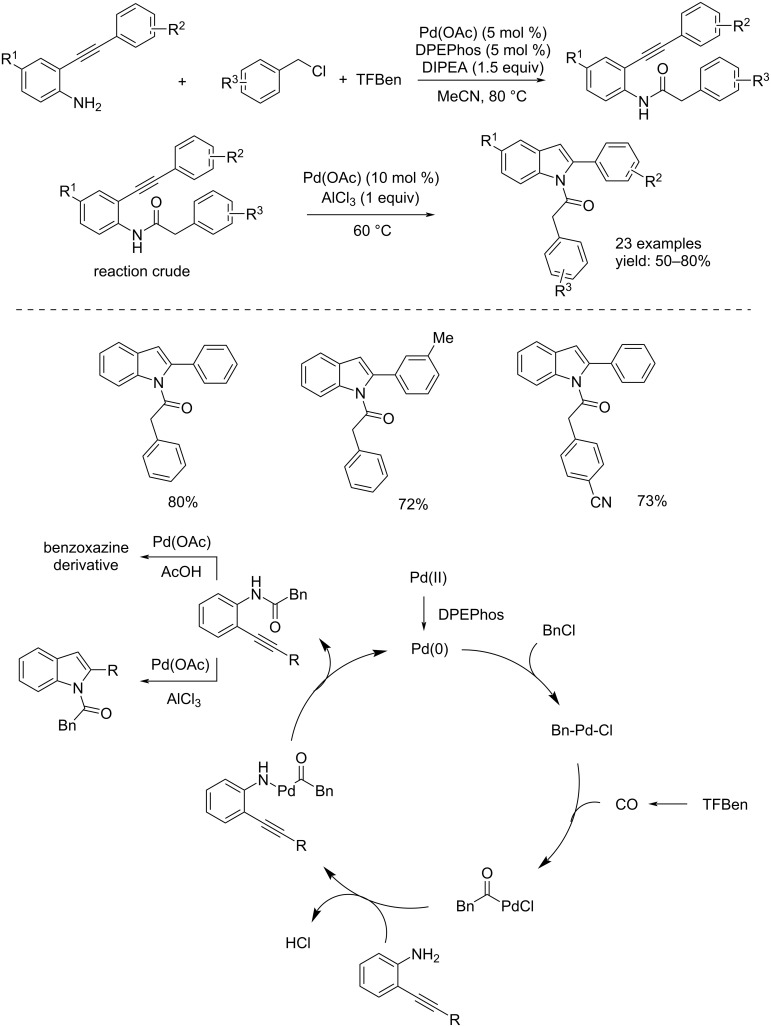
Pd(0)-catalyzed gas-free carbonylation of 2-alkynylanilines to 1-(1*H*-indol-1-yl)-2-arylethan-1-ones.

Instead, in the Senadi et al. approach, 1-(3-amino)-1*H*-indol-2-yl)-1-ketones were obtained through a Pd(0)-catalyzed cascade process consisting of isonitrile insertion as carbon monoxide surrogate and a C–H cross-coupling [13]. The reaction took place in the presence of K_2_CO_3_ (3 equiv) as base, an isonitrile (1.2 equiv), and Pd(OAc)_2_ (10 mol %) which in situ undergoes a reduction to Pd(0) (Scheme 2). Another example was published by Wu's group, who carried out the synthesis of 1-(1*H*-indol-1-yl)-2-arylethan-1-one derivatives by promoting the formation of amides from 2-alkynylanilines by using TFBen (benzene-1,3,5-triyl triformate) as a CO source, Pd(OAc)_2_, DPEPhos (bis[(2-diphenylphosphino)phenyl] ether), and DIPEA (*N*,*N*-diisopropylethylamine) in MeCN. After 24 h, Pd(OAc)_2_ and AlCl_3_ were added to promote a selective cyclization reaction [14]. The reaction mechanism proceeds with an initial reduction of Pd(II) to Pd(0) followed by oxidative addition on the ArCH_2_–Cl bond to form the ArCH_2_–Pd^II^–Cl complex. Then, insertion of CO, from TFBen, takes place followed by nucleophilic displacement and reductive elimination. The obtained compound undergoes selective cyclization to the indole derivative in the presence of Pd(OAc)_2_ and AlCl_3_. A variety of indole derivatives were synthetized in good isolated yields (Scheme 3).

#### Synthesis of indoles by Pd(II)-catalyzed carbonylation reaction

**Scheme 4:**
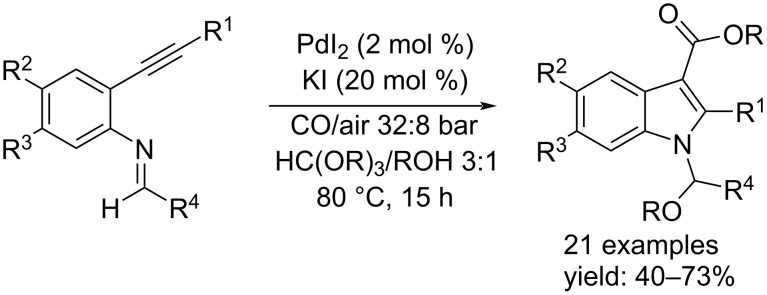
Pd(II)-catalyzed heterocyclization/alkoxycarbonylation of 2-alkynylaniline imines.

Oxidative carbonylation reactions, as well as all other types of carbonylation reactions, represent a simple and more environmentally friendly method for the synthesis of important organic compounds. Since the development of the Wacker process, the study of oxidative carbonylations has made enormous progress by investigating even the possibility of catalyzing carbonylative heterocyclization reactions by exploiting the electrophilic character of Pd(II) species. Starting from substrates bearing a triple bond and a nucleophile in the appropriate position, a versatile process of heterocyclization can be initiated, resulting in indole derivatives with important properties. Gabriele et al., in two different periods, reported two paradigmatic examples for indole syntheses. In 2010, they developed the synthesis of 1-(alkoxyarylmethyl)indole-3-carboxylic esters from 2-alkynylaniline imines by using PdI_2_/KI as catalyst system and oxygen as oxidant [15]. In particular, the reaction was carried out using 2 mol % of PdI_2_, 20 mol % of KI and 40 bar of a 4:1 mixture of CO–air, in methanol or ethanol in the presence of an *ortho*-ester (1:3 mixture) as solvent to prevent hydrolysis of the substrate. After 15 hours at 80 °C the indoles derivatives were isolated in good yields (Scheme 4).

**Scheme 5:**
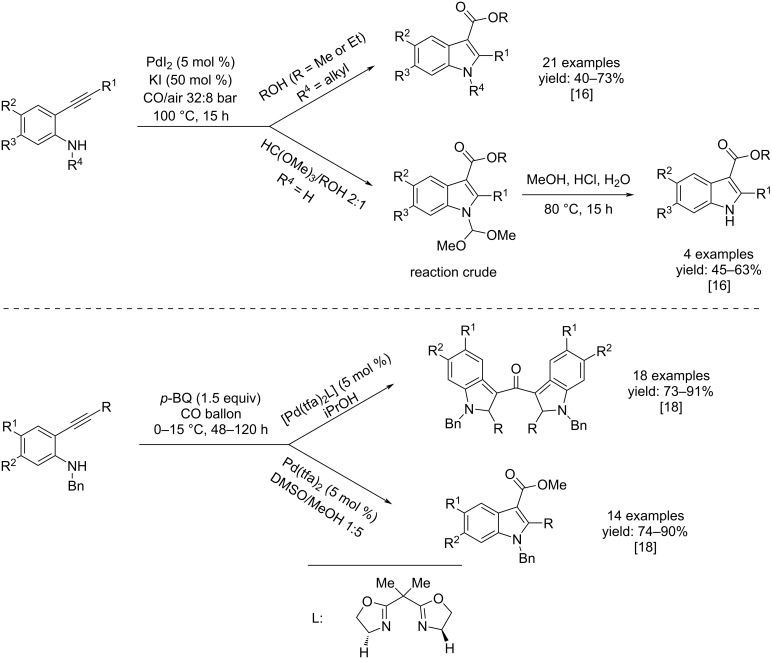
Pd(II)-catalyzed heterocyclization/alkoxycarbonylation of 2-alkynylanilines to *N*-substituted indole-3-carboxylic esters and indole-3-carboxylic esters (top). Pd(II)-catalyzed selective carbonylation of 2-alkynylaniline to methyl 1-benzyl-1*H*-indole-3-carboxylates and bis(1-benzyl-1*H*-indol-3-yl)methanones (bottom).

Two years later, they performed, using the same catalytic and oxidative conditions, another oxidative heterocyclization/alkoxycarbonylation process for the synthesis of *N*-substituted indole-3-carboxylic esters and N–H free indole-3-carboxylic esters from *N*-substituted 2-alkynylanilines and 2-alkynylanilines bearing a secondary amino group and an internal triple bond [16]. In the same research group, it has been demonstrated that with a terminal alkynyl group, the reactivity is completely different leading to the formation of dihydroindol-2-one derivatives [17]. The process was versatile and efficient towards the formation of *N*-substituted indole derivatives from *N*-substituted anilines. The direct PdI_2_/KI oxidative carbonylation of 2-alkynylanilines does not lead to the formation of indole-3-carboxylic esters but to the formation of acyclic carbamates. For this reason, they performed the reaction in the presence of trimethyl orthoformate to transform, in situ, the primary amino group into a secondary amino group with an easy-to-remove substituent thus performing a not direct synthesis process. Indeed, the desired N–H-free product was obtained by acidic treatment of the reaction crude. In both cases, the targeted products were isolated in good yields (Scheme 5).

In 2014, Shen and co-workers developed a selective synthesis for methyl 1-benzyl-1*H*-indole-3-carboxylates and bis(1-benzyl-1*H*-indol-3-yl)methanones [18] starting from the same kind of substrates used by Gabriele’s group two years before. The first indole derivatives were obtained by catalyzing the reaction with 5 mol % of Pd(tfa)_2_ (palladium(II) trifluoroacetate) and 1.5 equivalents of *p*-benzoquinone as oxidant in a 1:5 DMSO/MeOH solvent mixture at a temperature between 0 °C and 15 °C and, for a time between 48 and 120 hours depending on the substrate. In the second case the reaction was catalyzed under the same conditions except for changing the solvent to iPrOH and the catalyst to [Pd(tfa)_2_L] (Scheme 5).

**Scheme 6:**
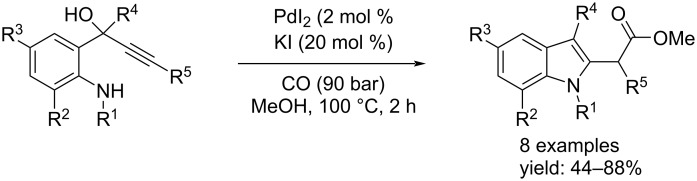
Synthesis of indol-2-acetic esters by Pd(II)-catalyzed carbonylation of 1-(2-aminoaryl)-2-yn-1-ols.

Furthermore, Gabriele and co-workers developed the oxidative carbonylation of 1-(2-aminoaryl)-2-yn-1-ols to quinoline-3-carboxylic esters. Meanwhile, they also discovered that by conducting the reaction under non-oxidative conditions the reaction mechanism changed, leading to the formation of indol-2-acetic esters via the H–Pd^II^–I species formed in situ [19]. The reaction was performed in the presence of PdI_2_ and KI (2 mol % and 20 mol %, respectively) in methanol under 90 bar of CO at 100 °C for two hours (Scheme 6).

**Scheme 7:**
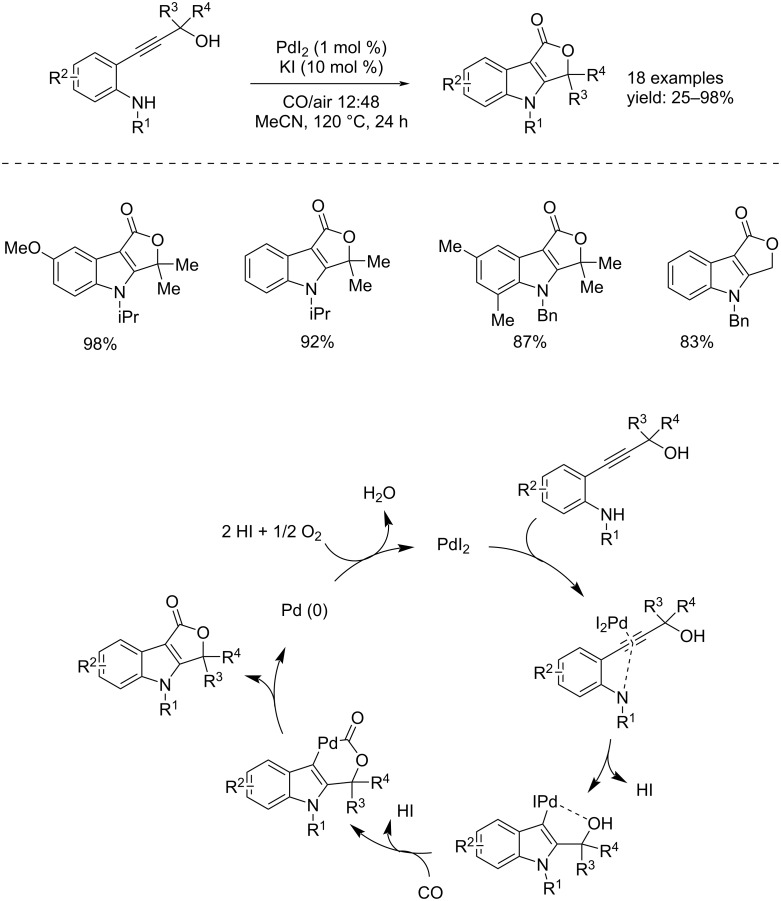
Pd(II)-catalyzed carbonylative double cyclization of suitably functionalized 2-alkynylanilines to 3,4-dihydro-1*H*-furo[3,4-*b*]indol-1-ones.

As already seen, triple bonds can be activated by Pd(II) catalysts towards the addition of nucleophiles in the right position, leading to heterocyclization reactions. Taking advantage of this possibility, in the Della Cá group, a carbonylative double cyclization process was developed obtaining 3,4-dihydro-1*H*-furo[3,4-*b*]indol-1-ones from suitably functionalized 2-alkynylanilines [20]. The reaction occurs in the presence of PdI_2_ (1 mol %) as catalyst and KI (10 mol %) as co-catalyst in MeCN at 120 °C for 24 h. At the end of the process the catalyst undergoes reduction, therefore, rather using only CO, a mixture of CO–air (12:48 bar) was used with the aim of oxidizing the Pd(0) species in order to restore the catalyst able to catalyze the process again. The reaction mechanism proceeds with an initial interaction between the Pd(II) species and the triple bond that promotes an intramolecular nucleophilic attack of the amino group giving a indolcyclopalladium species. This is followed by CO insertion and intramolecular nucleophilic displacement by the hydroxy group to give the indole–Pd^II^-cycle derivate. The reaction ends with a reductive elimination and the generated Pd(0) species gets oxidated by the oxygen to the active Pd(II) species (Scheme 7).

#### Synthesis of indoles by metal-catalyzed reductive cyclization reaction of organic nitro compounds with carbon monoxide as reductant

**Scheme 8:**
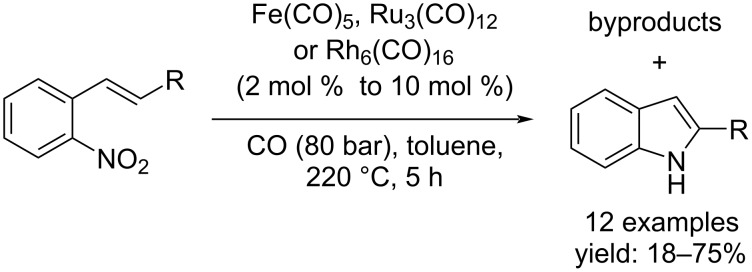
Indole synthesis by deoxygenation reactions of nitro compounds reported by Cenini et al. [21].

In the last 60 years, the metal-catalyzed carbonylative reduction approach of organic nitro compounds has been used for the synthesis of important industrial and pharmaceutical compounds such as indoles. In 1986, Cenini et al. reported a new synthetic method for indoles that involved 2-nitrostyrene derivatives. The reaction took place under drastic conditions (200 °C and 80 bar CO) for 5 hours in the presence of catalysts such as Fe(CO)_5_, Ru_3_(CO)_12_, or Rh_6_(CO)_16_ [21]. The process was not selective because aniline derivatives and other byproducts were also formed; moreover, the substrate conversion, in some cases, was not complete (Scheme 8).

**Scheme 9:**
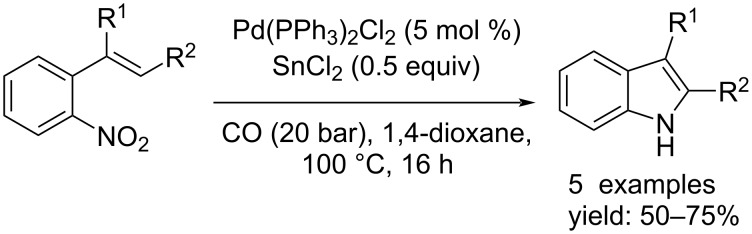
Indole synthesis by reduction of nitro compounds: approach reported by Watanabe et al. [22].

In subsequent years, this kind of synthesis was investigated by other groups. Watanabe's group improved the process by studying the reactivity of Pd(PPh_3_)_2_/SnCl_2_ as a new catalyst system [22]. This made it possible to conduct the reaction under milder conditions, making the process less expensive and more environmentally friendly. Indeed, the reaction was carried out under 20 bar of CO at 100 °C for 16 h. The indole derivatives were obtained in moderate to good yields and complete conversion of the substrates was observed (Scheme 9).

**Scheme 10:**
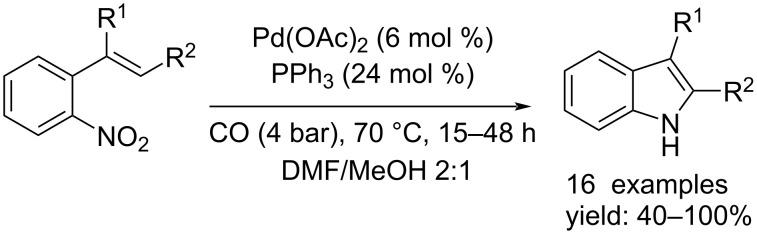
Indole synthesis from *o*-nitrostyrene compounds as reported by Söderberg and co-workers [23].

In 1997, Söderberg and co-workers reported a more sustainable procedure. In this protocol the use of a Lewis base such as SnCl_2_ was not required and the reaction took place under milder conditions using Pd(OAc)_2_ as catalyst and PPh_3_ as ligand. The reaction was performed at 70 °C for 15–48 hours, under 4 bar of CO in a DMF/MeOH 2:1 mixture giving the indole derivatives without obvious byproducts (Scheme 10) [23].

**Scheme 11:**
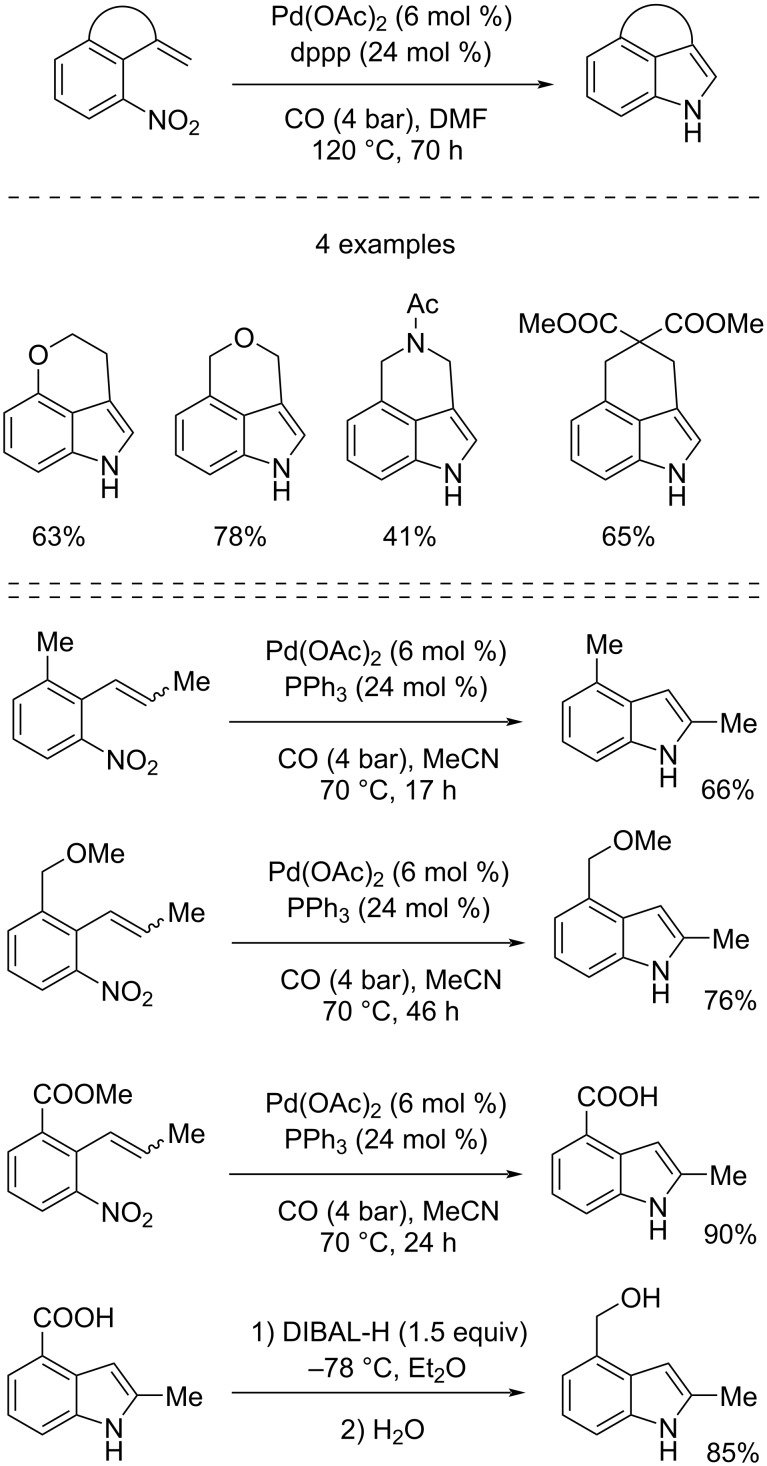
Synthesis of fused indoles (top) and natural indoles present in two species of *European Basidiomycetes* (bottom) by reductive *N*-heteroannulation of suitably functionalized nitro compounds.

Two years later they developed two Pd-catalyzed reductive *N*-heteroannulations for the synthesis of fused indoles [24] and indoles isolated from two species of *European Basidiomycetes* [25] starting from suitably functionalized nitro compounds. Both in the first case and in the second case the reactions were catalyzed by 6 mol % of Pd(OAc)_2_ and carried out under 4 bar of CO. The fused indoles were obtained when adding 24 mol % of dppp (1,3-bis(diphenylphosphino)propane) at 120 °C for 70 hours in DMF. Instead, in the other case, PPh_3_ was used as ligand at 70 °C for 17–46 hours in CH_3_CN (Scheme 11).

**Scheme 12:**
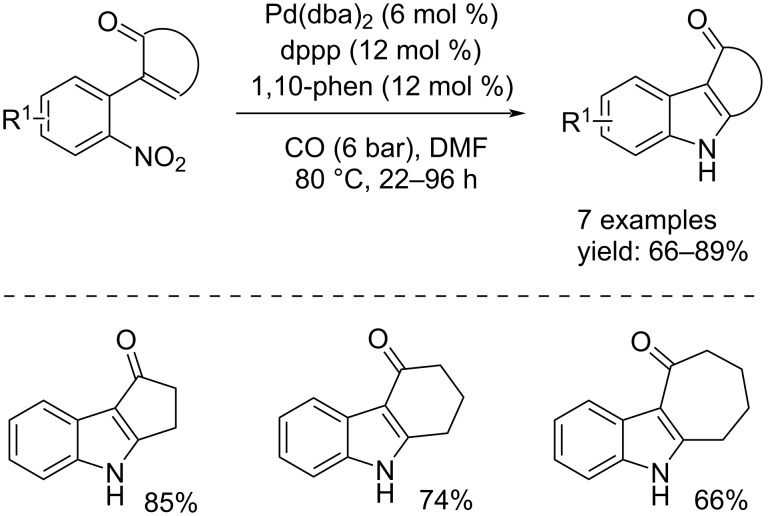
Synthesis of 1,2-dihydro-4(3*H*)-carbazolones through *N*-heteroannulation of functionalized 2-nitrostyrenes in the presence of the Pd(dba)_2_/dppp/1,10-phen catalyst system.

Another interesting example concerns the synthesis of 1,2-dihydro-4(3*H*)-carbazolones by a Pd(0)-catalyzed *N*-heteroannulation of functionalized 2-nitrostyrenes [26]. Pd(dba)_2_ was able to catalyze the reaction because a five-membered Pd(II)-cyclic species could be formed, favored by the oxidation of the catalyst. The reaction proceeded with dppp and 1,10-phenantroline (12 mol % each) under 6 bar of CO at 80 °C in DMF for 22–96 hours depending on the substrate. After the required reaction time, seven carbazolones were isolated with good yields of up to 89% (Scheme 12).

**Scheme 13:**
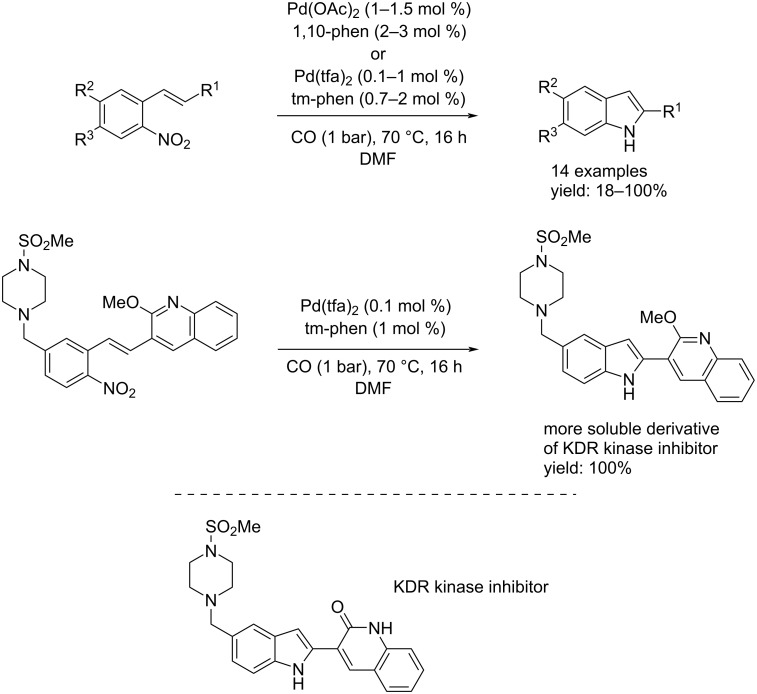
Synthesis of indoles from *o*-nitrostyrenes by using Pd(OAc)_2_ and Pd(tfa)_2_ in conjunction with bidentate nitrogen ligands: 1,10-phen (1,10-phenanthroline) and tm-phen (3,4,7,8-tetramethyl-1,10-phenanthroline).

In 2005, Davies et al. published a paper presenting a new method for the synthesis of indoles from *o*-nitrostyrenes by using a different catalyst system and performing the reaction under mild conditions [27]. At first they decided to change the catalytic system applied by Söderberg using 1,10-phen instead of PPh_3_ as ligand, because it was already known that catalysts derived from palladium(II) salts and bidentate nitrogen ligands were highly reactive systems for the reduction of *o*-nitrostyrenes [28–30]. The catalytic system Pd(OAc)_2_/1,10-phen worked better than Söderberg´s one (Pd(OAc)_2_/PPh_3_) under mild conditions. In addition, Pd(phen)_2_(BF_4_)_2_ and Pd(tfa)_2_ in conjunction with tertramethyl-1,10-phenanthroline (tm-phen) reactivity were investigated. The process was generalized: by using Pd(OAc)_2_ (1–1.5 mol %) and 1,10-phen (2–3 mol %) to catalyze the reaction of some substrates and Pd(tfa)_2_ (0.1–1 mol %) and tm-phen (0.7–2 mol %) for others, 14 indole derivatives were isolated with yields of up to 100%. Among the 14 examples is a more soluble derivative of a KDR kinase inhibitor identified by researchers from Merck for blocking tumor-induced angiogenesis [31] (Scheme 13).

An interesting synthesis of 3-arylindoles by reductive carbonylation of unfunctionalized nitroarenes in the presence of arylalkynes and Pd(phen)_2_(BF_4_)_2_ as catalyst has been demonstrated by Cenini's group. The reaction was regioselective, with no detection of 2-arylindole byproducts. No prefunctionalization of the *ortho* position of the nitroarene is required for the reaction [32]. When Pd(phen)_2_(BF_4_)_2_ was used in conjunction with Ru_3_(CO)_12_ the yield of the indole products increased. Additionally, when 4-fluorophenylacetylene and nitrobenzene were used as substrates the indole skeleton of fluvastatin and other pharmaceutically active compounds was obtained in one step [33].

**Scheme 14:**
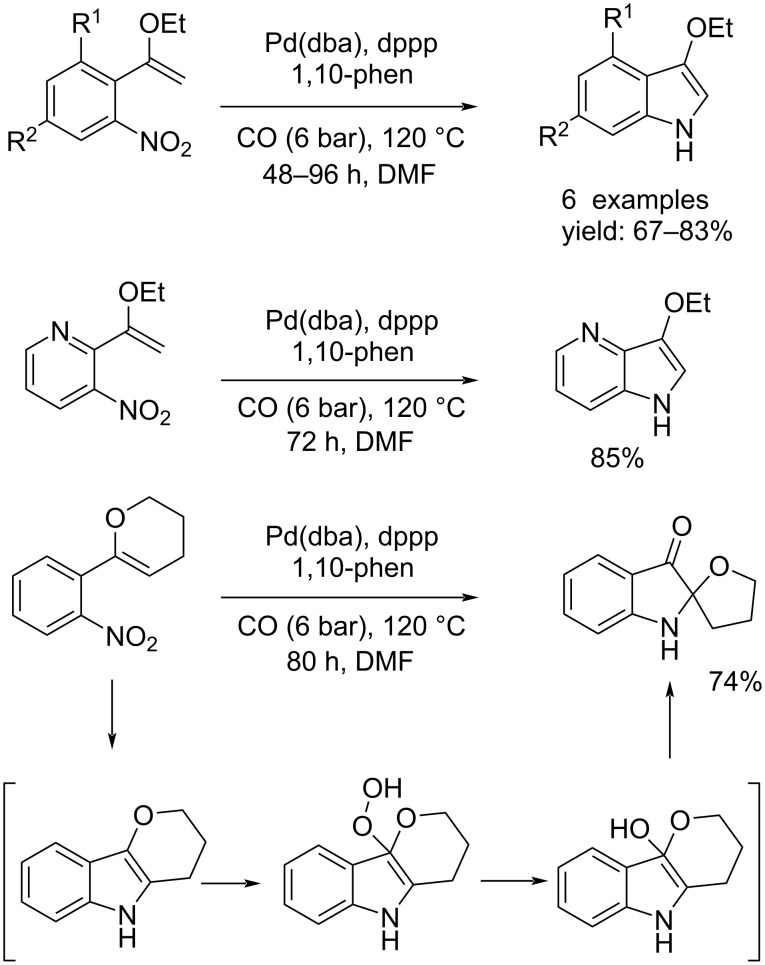
Synthesis of substituted 3-alkoxyindoles via palladium-catalyzed reductive *N*-heteroannulation.

Clawson et al. showed that it was possible to achieve the synthesis of substituted 3-alkoxyindoles via the palladium-catalyzed reductive *N*-heteroannulation of 1-(2-nitrophenyl)-1-alkoxyalkenes. Only in one case 2-(1-ethoxyvinyl)-3-nitropyridine was used [34]. The reactions were catalyzed through Pd(dba)_2_, dppp, 1,10-phenantroline under 6 bar of CO at 120 °C in DMF and the products were obtained within 48–96 hours. All products were isolated with good yields except the pyranindole because it decomposed; it could only be isolated after complete oxidation in air. The isolated product was an indol-3-one derivative (Scheme 14).

**Scheme 15:**
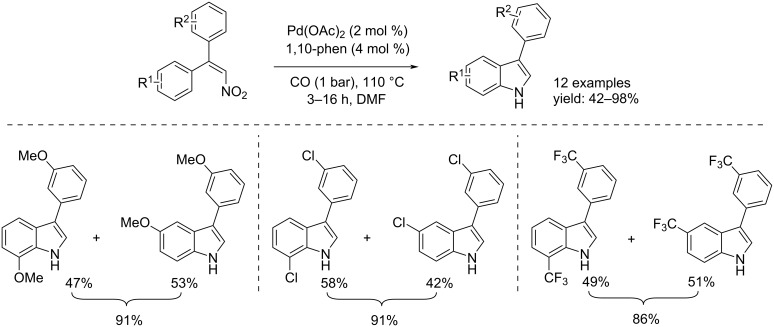
Synthesis of 3-arylindoles by palladium-catalyzed C–H bond amination via reduction of nitroalkenes.

Another example for the synthesis of 3-substituted indoles was described by Hsieh and Dong [35]. They synthesized 3-arylindoles by palladium-catalyzed C–H bond amination via reduction of nitroalkenes using carbon monoxide as reducing agent. The reaction took place in the presence of Pd(OAc)_2_ (2 mol %), 1,10-phenantroline (4 mol %) under 1 bar of CO at 110 °C in DMF. After the right time, six products were isolated, while, in three cases, when the benzene ring was *meta*-substituted, a regioisomeric mixture was obtained. The regioselectivity was determined by ^1^H NMR spectroscopy (Scheme 15).

**Scheme 16:**
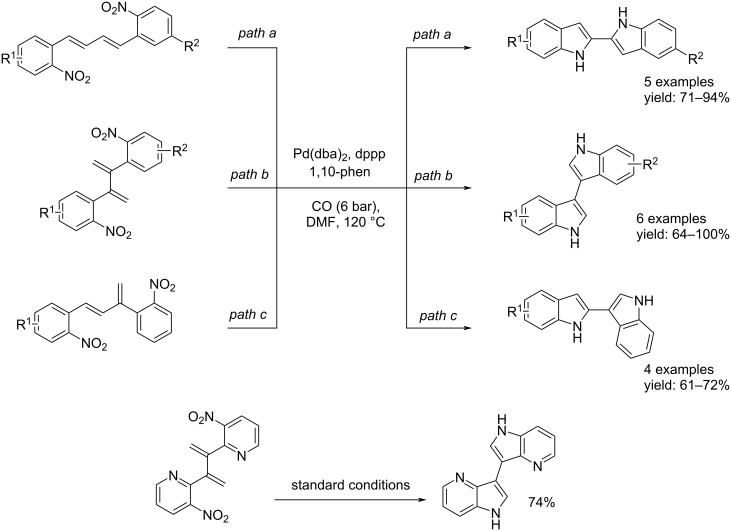
Synthesis of 2,2′-bi-1*H*-indoles, 2,3′-bi-1*H*-indoles, 3,3′-bi-1*H*-indoles, indolo[3,2-*b*]indoles, indolo[2,3-*b*]indoles, and 4,4′-diaza-3,3′-bi-1*H*-indole.

**Scheme 17:**
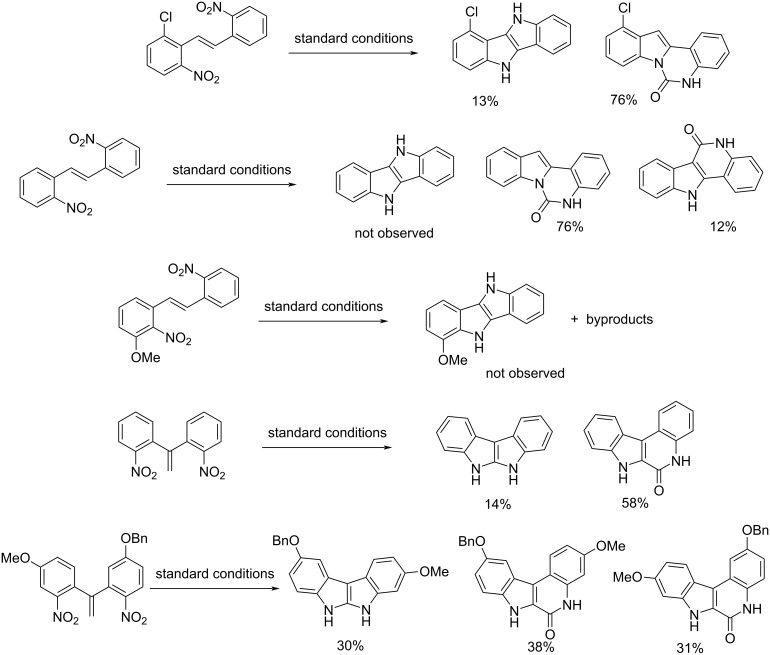
Pd-catalyzed reductive cyclization of 1,2-bis(2-nitrophenyl)ethene and 1,1-bis(2-nitrophenyl)ethene derivatives.

More recently, in 2016, still in Söderberg’s group, the synthesis of 2,2′-bi-1*H*-indoles, 2,3′-bi-1*H*-indoles, 3,3′-bi-1*H*-indoles, indolo[3,2-*b*]indoles, and indolo[2,3-*b*]indoles via reductive cyclization Pd-catalyzed was developed [36]. The reaction was possible starting from nitro compounds with one or two double bonds in the suitable position. The process has been generalized using nitro compounds substituted on the aromatic ring with electron-donating and electron-withdrawing groups. Also the synthesis of 4,4′-diaza-3,3′-bi-1*H*-indole from 2,2'-(buta-1,3-diene-2,3-diyl)bis(3-nitropyridine) was performed. All reactions were carried out in the presence of Pd(dba)_2_ as catalyst, dppp and 1,10-phen as ligands under 6 bar of CO at 120 °C for the appropriate time. The processes to 2,2′-bi-1*H*-indoles, 2,3′-bi-1*H*-indoles, and 3,3′-bi-1*H*-indoles were selective leading to product formation with good to excellent isolated yields (Scheme 16). On the other hand, the synthesis of indolo[3,2-*b*]indoles and indolo[2,3-*b*]indoles were not selective leading to byproducts such as 5,7-dihydro-6*H*-indolo[2,3-*c*]quinolin-6-one, 5,11-dihydro-6*H*-indolo[3,2-*c*]quinolin-6-one, and indolo[1,2-*c*]quinazolin-6(5*H*)-one derivatives. Finally, starting from 1,2-bis(2-nitrophenyl)ethene and 1-methoxy-2-nitro-3-(2-nitrostyryl)benzene the related indolo[3,2-*b*]indoles were not observed (Scheme 17).

**Scheme 18:**
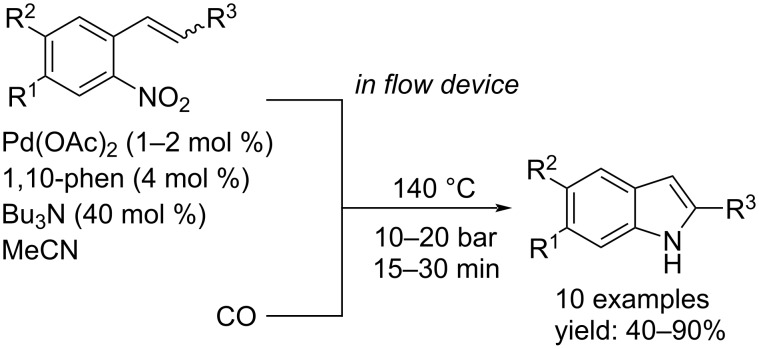
Flow synthesis of 2-substituted indoles by reductive carbonylation.

One year later, by using continuous flow technology, Gutmann, Kappe and colleagues developed a palladium-catalyzed transformation of *o*-vinylnitrobenzenes and *o*-nitrostilbenes with carbon monoxide as the reductant to give indoles after cyclization [37]. In the presence of 1–2 mol % of Pd(OAc)_2_, 1,10-phen (4 mol %) and tributylamine (40 mol %), the desired products were formed in good to excellent yields in CH_3_CN under 10–20 bar of CO at 140 °C within 15–30 min and with carbon dioxide as the side-product (Scheme 18).

**Scheme 19:**
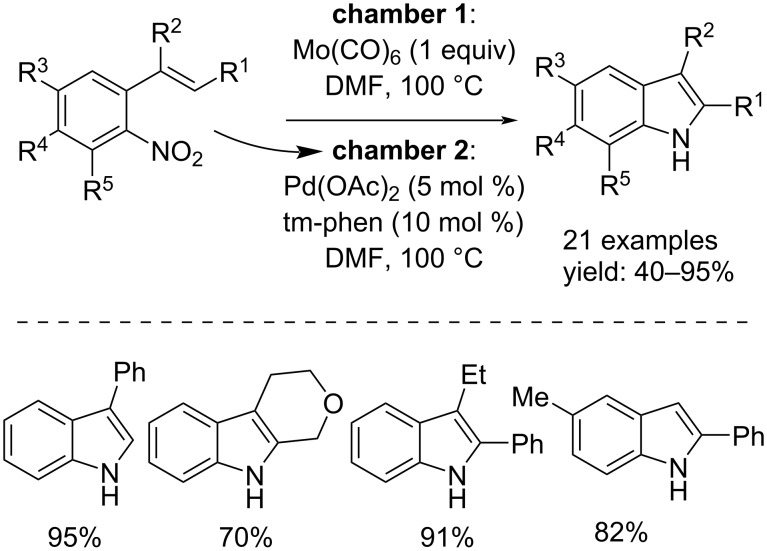
Pd-catalyzed synthesis of variously substituted 3*H*-indoles from nitrostyrenes by using Mo(CO)_6_ as CO source.

Carbon monoxide is a cheap gas but its use requires safe and controlled environments and systems because it is highly toxic to human. In addition, reactions in which carbon monoxide is used as a gas require suitable equipment to withstand the high pressures such as autoclaves. The use of compounds able to generate carbon monoxide in situ is a convenient, less dangerous and effective method to perform carbonylation reactions using less complex apparatus. In recent years, many syntheses via carbonylation reactions, without the use of carbon monoxide, have been demonstrated. Regarding the synthesis of indoles, Zhou et al. discovered that the combination of Mo(CO)_6_, Pd(OAc)_2_ and 1,10-phen, was suitable to afford 3*H*-indoles from variously substituted nitrostyrenes [38]. CO is generated by heating 1 equiv of Mo(CO)_6_ at 100 °C in DMF in a sealed glass chamber (chamber 1). Afterwards, the gas passes into another sealed glass chamber (chamber 2) containing the substrate, Pd(OAc)_2_ (5 mol %), tm-phen (10 mol %) and DMF at the same temperature (Scheme 19).

**Scheme 20:**
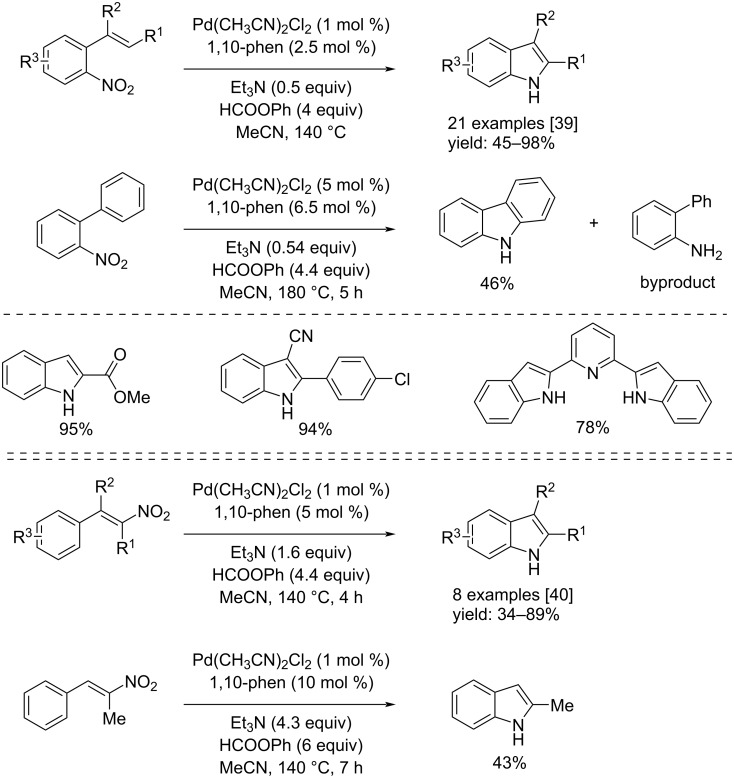
Synthesis of indoles from substituted 2-nitrostyrenes (top) and ω-nitrostyrenes (bottom) via reductive cyclization with phenyl formate as the carbon monoxide source.

**Scheme 21:**
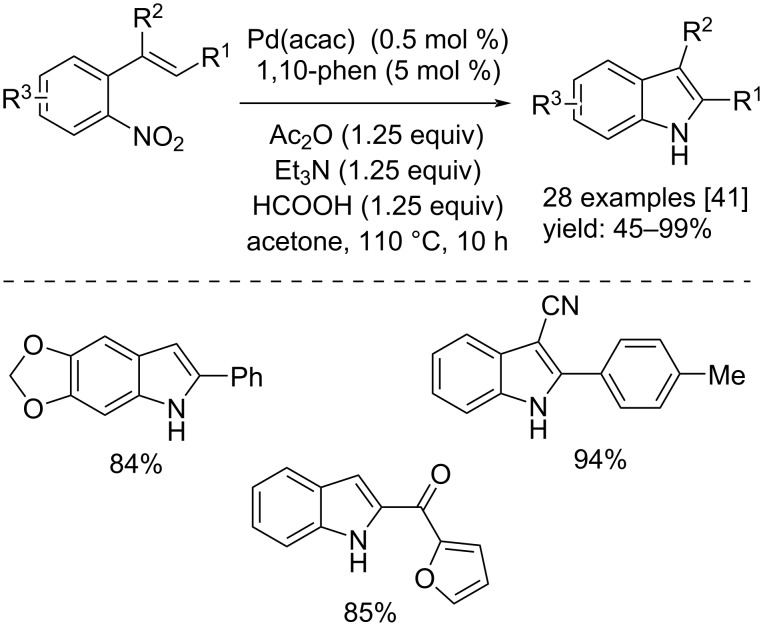
Synthesis of indoles from substituted 2-nitrostyrenes with formic acid as CO source.

Ragaini and co-workers, in 2018, 2022 and 2023, developed three procedures for the synthesis of substituted indoles from functionalized nitro compounds by using, in the first two cases phenyl formate as CO source [39–40] and, in the third case formic acid [41]. All of three processes allow the use of inexpensive reactors, in fact, the reactions were carried out in heavy borosilicate tubes. In the first two works, Pd(MeCN)_2_Cl_2_ in conjunction with 1,10-phen were used to catalyze the reactions. In addition, Et_3_N was added to the reaction mixture in order to favor the catalyst´s reduction to Pd(0). In the work published in 2022, some substrates led to a non-selective reaction (Scheme 20). On the other hand, in the approach in which formic acid was used, the catalyst system was Pd(acac)_2_/1,10-phen. Also, in this case the addition of Et_3_N favored the reaction, moreover, Ac_2_O was added as additive (Scheme 21).

**Scheme 22:**
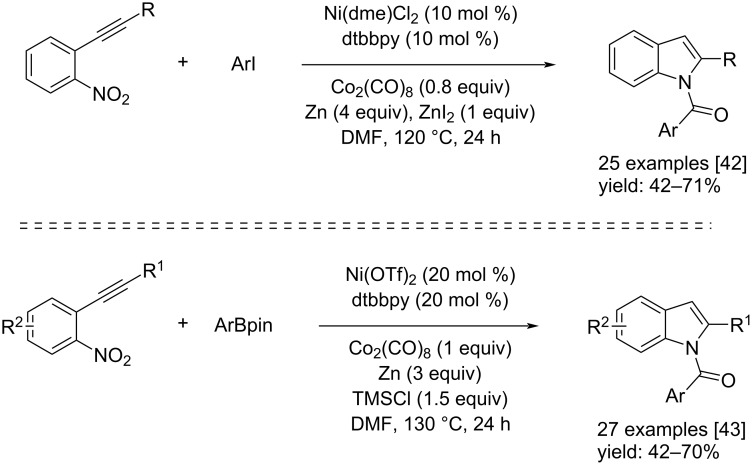
Ni-catalyzed carbonylative cyclization of 2-nitroalkynes and aryl iodides (top) and the Ni-catalyzed carbonylative cyclization of 2-nitroalkynes and arylboronic pinacol esters (bottom) reported by Wu and co-workers [42–43].

**Scheme 23:**
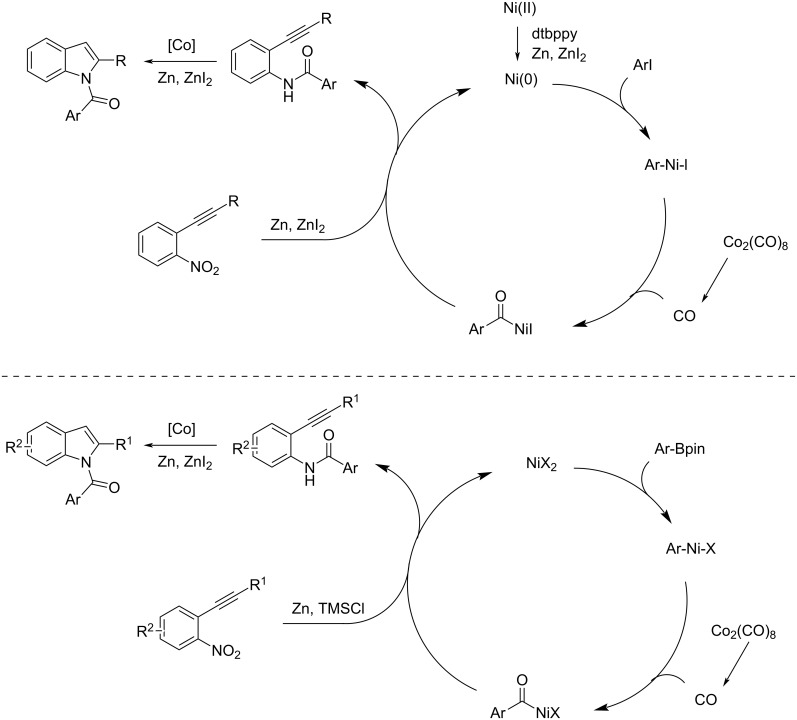
Mechanism of the Ni-catalyzed carbonylative cyclization of 2-nitroalkynes and aryl iodides (top) and Ni-catalyzed carbonylative cyclization of 2-nitroalkynes and arylboronic pinacol esters (bottom).

At the same time, Wu and co-workers contributed to the introduction of two new syntheses of *N*-aroylindole derivatives by means of nickel catalysis. In 2021, they reported a nickel-catalyzed carbonylative cyclization of 2-nitroalkynes and aryl iodides with Co_2_(CO)_8_ as the CO source. The reaction was performed in the presence of Ni(dme)Cl_2_ (a nickel(II) chloride ethylene glycol dimethyl ether complex), dtbbpy (4,4-di-*tert*-butyl-2,2-dipyridyl), Zn(0) and ZnI_2_ in DMF at 120 °C [42] (Scheme 22). The nickel catalyst catalyzed the oxidative addition and CO insertion on aryl iodide compounds, while the Zn/ZnI_2_ couple catalyzed the reduction of the nitro group with CO as reductant. The last step is an *N*-cyclization toward the 2-aroylindole formation with moderate to high isolated yields. In the other example, reported one year later, the same kind of compounds was obtained from 2-nitroalkynes and arylboronic pinacol esters [43]. The reaction took place in the presence of Ni(OTf)_2_, dtbbpy, Zn(0), and TMSCl (trimethylsilyl chloride) in DMF at 130 °C (Scheme 22). The Ni(OTf)_2_ catalyzed the transmetallation reaction with Ar-Bpin and subsequent insertion of CO. The reduction of the nitro group is catalyzed by Zn(0)/TMSCl in the presence of CO. The reaction, again, ends with an *N*-cyclization giving the *N*-aroylindoles with fair to good isolated yields. The reaction mechanisms are reported in Scheme 23.

#### Synthesis of indoles by Rh-catalyzed carbonylation reactions

**Scheme 24:**
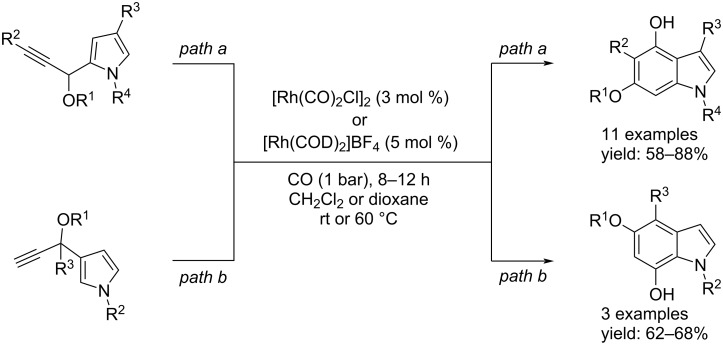
Route to indole derivatives through Rh-catalyzed benzannulation of heteroaryl propargylic esters favoured by CO.

In the past, Tang and co-workers, demonstrated that vinyl propargylic esters could be employed as five-carbon atom building blocks in [5 + 2] cycloadditions with alkynes or alkenes by carbene intermediates. Starting from those results they envisaged the benzannulation of heteroaryl propargylic esters favored by CO [44]. The process led to the desired result with some effort because a dearomatization, followed by aromatization, was necessary to achieve the goal. With [Rh(CO)_2_Cl]_2_ or [Rh(COD)_2_]BF_4_ as the catalyst under atmospheric pressure of CO (1 bar), good yields of the desired products were obtained (Scheme 24).

### Carbonylative functionalization of indoles

#### Metal-catalyzed cyclocarbonylative coupling reaction of indoles to 6*H*-isoindolo[2,1-*a*]indol-6-one scaffolds

**Scheme 25:**
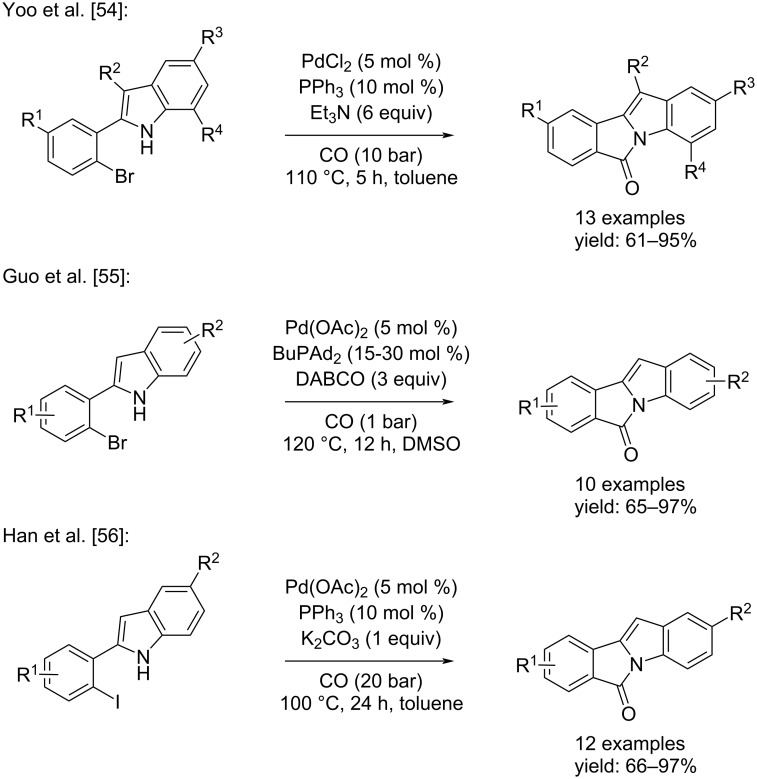
Pd-catalyzed cyclization of 2-(2-haloaryl)indoles reported by Yoo and co-workers [54], Guo and co-workers [55], and Han and co-workers [56] for the synthesis of 6*H*-isoindolo[2,1-*a*]indol-6-ones.

Substituted 6*H*-isoindolo[2,1-*a*]indol-6-ones are important structural components of many naturally occurring and pharmacologically active compounds [45–51]. They are also relevant intermediates in organic synthesis [52–53]. Therefore, the significance of 6*H*-isoindolo[2,1-*a*]indol-6-ones has led to a longstanding interest in the development of efficient and versatile methods for their synthesis. In 2016, four independent studies reported the first successful application of a metal-catalyzed cyclocarbonylation in an efficient synthetic pathway towards 6*H*-isoindolo[2,1-*a*]indol-6-ones. In three of these, the products were obtained through Pd-catalyzed cyclization of 2-(2-haloaryl)indoles: Yoo et al. reported the synthesis by using various 2-(2-bromophenyl)-1*H*-indoles, PdCl_2_/PPh_3_ as catalyst system in the presence of a base (Et_3_N) in toluene at 110 °C for 5 hours under 10 bar of CO [54]. Guo et al. developed the synthesis from substrates of the same chemical nature but using Pd(OAc)_2_/BuPAd_2_ as the catalyst system. In their approach, DABCO was also added to the reaction mixture in DMSO and the reaction was carried out at 120 °C for 12 hours under a low pressure of CO (1 bar) [55]. Another version was reported by Han and co-workers, who synthesized the desired products starting from 2-(2-iodophenyl)-1*H*-indoles, catalyzing the reaction with Pd(OAc)_2_, PPh_3_, and K_2_CO_3_. The reaction was run in toluene at 100 °C for 24 h under 20 bar of CO [56] (Scheme 25).

**Scheme 26:**
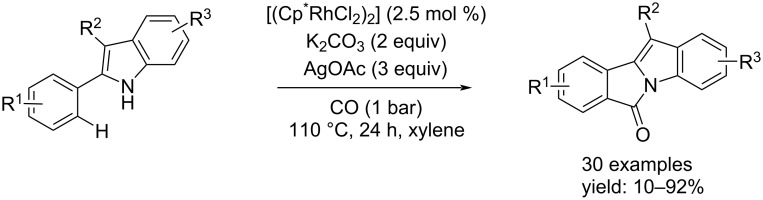
Approach for the synthesis of 6*H*-isoindolo[2,1-*a*]indol-6-ones reported by Huang and co-workers [57].

The other example, however, accomplished the synthesis through Rh-catalysis from substrates without halogens in their structure. This synthesis was published by Huang et al. who obtained good results by using [(Cp*RhCl_2_)_2_] as catalyst to achieve an NH-indole–C–H carbonylation [57]. A base was added to improve the efficiency of the process and an oxidant to restore the catalytic active species. The reaction was carried out under a low pressure of CO (1 bar) at 110 °C in xylene for 24 hours (Scheme 26).

**Scheme 27:**
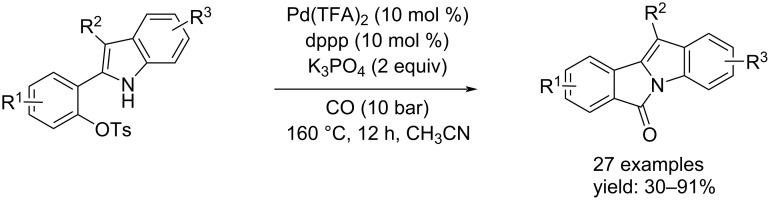
Zhou group’s method for the synthesis of 6*H*-isoindolo[2,1-a]indol-6-ones.

In 2018, the Zhou et al. proposed the synthesis of 2-(1*H*-indol-2-yl)phenyl tosylates via a Pd-catalyzed cycloaminocarbonylation reaction [58]. In this approach Pd(TFA)_2_/dppp was the catalyst system and able to catalyze the reaction in the best way. Also in this case a base was added to improve the reaction under 10 bar of CO, in CH_3_CN at 160 °C. By this route, 29 examples were synthesized with isolated yields up to 91% (Scheme 27).

**Scheme 28:**
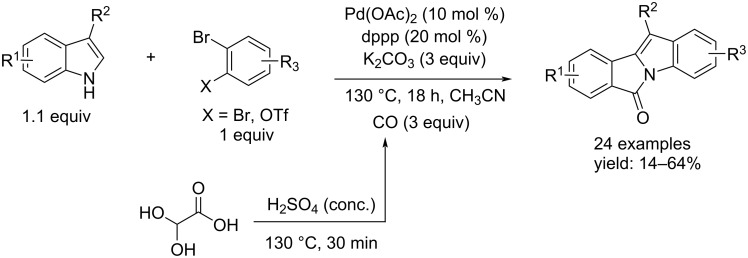
Synthesis of 6*H*-isoindolo[2,1-*a*]indol-6-ones from *o*-1,2-dibromobenzene and indole derivatives by using glyoxylic acid monohydrate as CO surrogate.

One year later, Čarný and co-workers described a facile construction of the isoindolo[2,1-*a*]indol-6-one structure via a Pd-catalyzed aminocarbonylation and C–H activation reaction starting from indoles and *o-*dibromoarenes as substrates [59]. In this tandem reaction, various symmetrical bromoarenes were utilized to eliminate the problem with regioselectivity. Two tests by using 2-bromo-5-methoxyphenyl triflate and 2-bromo-4-methoxyphenyl triflate were performed to evaluate the regioselectivity of the process. The regioselectivity was good with a regioisomeric ratio of 95:5. All reactions took place in the presence of Pd(OAc)_2_ as catalyst, cataCXium as ligand, and K_2_CO_3_ as base. Besides, the authors proposed glyoxylic acid monohydrate as an environmentally friendly CO surrogate (Scheme 28).

#### Metal-catalyzed carbonylative cyclization reaction of indole derivatives

**Scheme 29:**
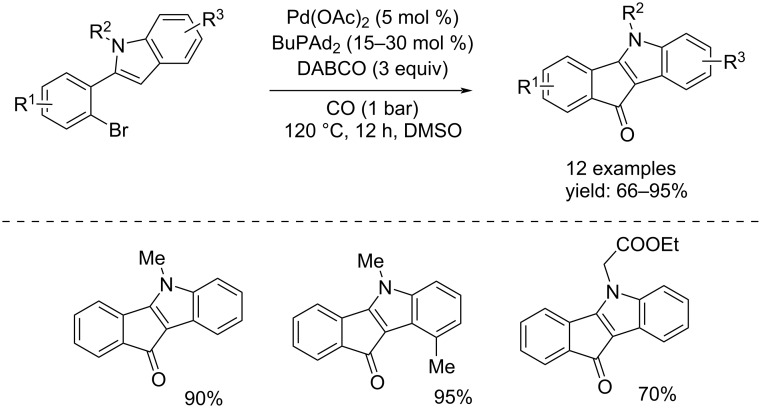
Pd(OAc)_2_-catalyzed Heck cyclization of 2-(2-bromophenyl)-1-alkyl-1*H*-indoles reported by Guo et al. [55].

In 2016, Guo and co-workers, in the same work in which different examples of 6*H*-isoindolo[2,1-*a*]indol-6-ones were reported, performed the synthesis of indeno[1,2-*b*]indol-10(5*H*)-ones from 2-(2-bromophenyl)-1-alkyl-1*H*-indoles [55] under the same conditions as mentioned in Scheme 25. The presence of an alkyl group on the indole nitrogen led to a Heck cyclization instead of a cycloaminocarbonylation (Scheme 29).

**Scheme 30:**
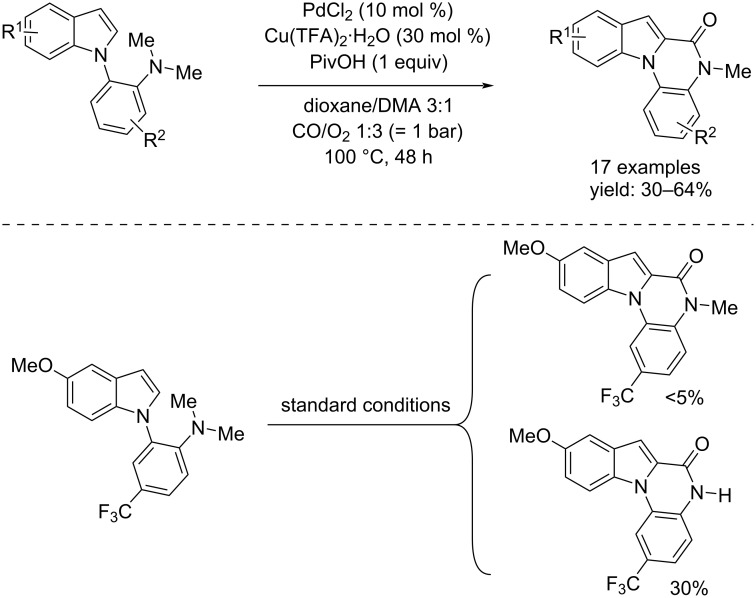
Synthesis of indolo[1,2-*a*]quinoxalinone derivatives through Pd/Cu co-catalyzed carbonylative cyclization from *o*-indolyl-*N*,*N*-dimethylarylamines.

In 2019, two different groups independently reported a carbonylative cyclization of *o*-indolylarylamines. In particular, Xu and co-workers used tertiary amines for achieving the reaction through a Pd/Cu co-catalysis [60]. The 17 examples were obtained by using PdCl_2_ (10 mol %) as catalyst and Cu(TFA)_2_∙H_2_O (30 mol %) as co-catalyst. At the end of the process the catalyst underwent reduction, and therefore, in order to make the process catalytic, the reaction was performed in the presence of O_2_. Furthermore, the addition of 1 equivalent of PivOH led to an improved isolated yield. A mixture of dioxane/DMA was the best solution to carry out the reaction that has been performed at 100 °C for 48 h (Scheme 30).

**Scheme 31:**
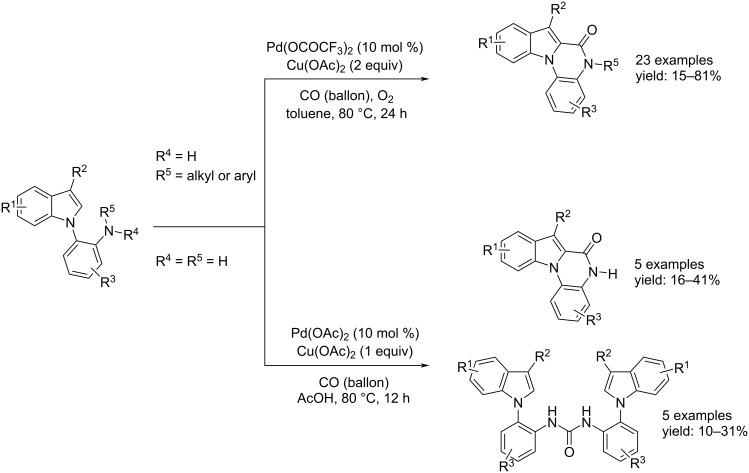
Pd-catalyzed carbonylative cyclization of *o*-indolylarylamines and *N*-monosubstituted *o*-indolylarylamines reported by Chandrasekhar and Sankararaman [61].

On the other hand, Chandrasekhar and Sankararaman reported the same process starting from secondary amines instead of tertiary amines [61]. In this case, the catalyst system was Pd(OCOCF_3_)_2_ (10 mol %) and the oxidant Cu(OAc)_2_ (2 equiv) and oxygen. The reaction was carried out in toluene at 80 °C for 24 h and, in terms of gas pressure, took place under mild conditions comparable to the ones reported by Xu et al. (Scheme 31). Chandrasekhar and Sankararaman also tested primary amines under the conditions outlined in Scheme 31, however, the reaction gave the annulated products (as the secondary amines) and the corresponding urea derivatives as byproducts.

**Scheme 32:**
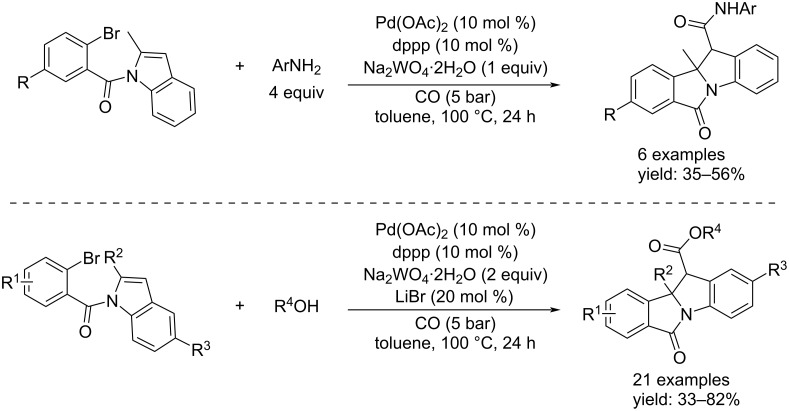
Pd-catalyzed diasteroselective carbonylative cyclodearomatization of *N*-(2-bromobenzoyl)indoles with anilines (top) and alcohols (bottom).

In the middle of the same year, Wu’s group succeeded in using *N*-(2-bromobenzoyl)indoles in the reaction with alcohols and anilines to achieve a Pd-catalyzed diasteroselective carbonylative cyclodearomatization [62]. The synthesis proceeded best when the reaction was performed in the presence of 10 mol % of Pd(OAc)_2_ and dppp as catalyst system, Na_2_WO_4_∙2H_2_O (1 or 2 equiv) as base and an excess of nucleophile (alcohols or anilines) in toluene as reaction solvent under 5 bar of CO at 100 °C. Also, 20 mol % of LiBr were added as additive when alcohols were used as the nucleophiles (Scheme 32).

**Scheme 33:**
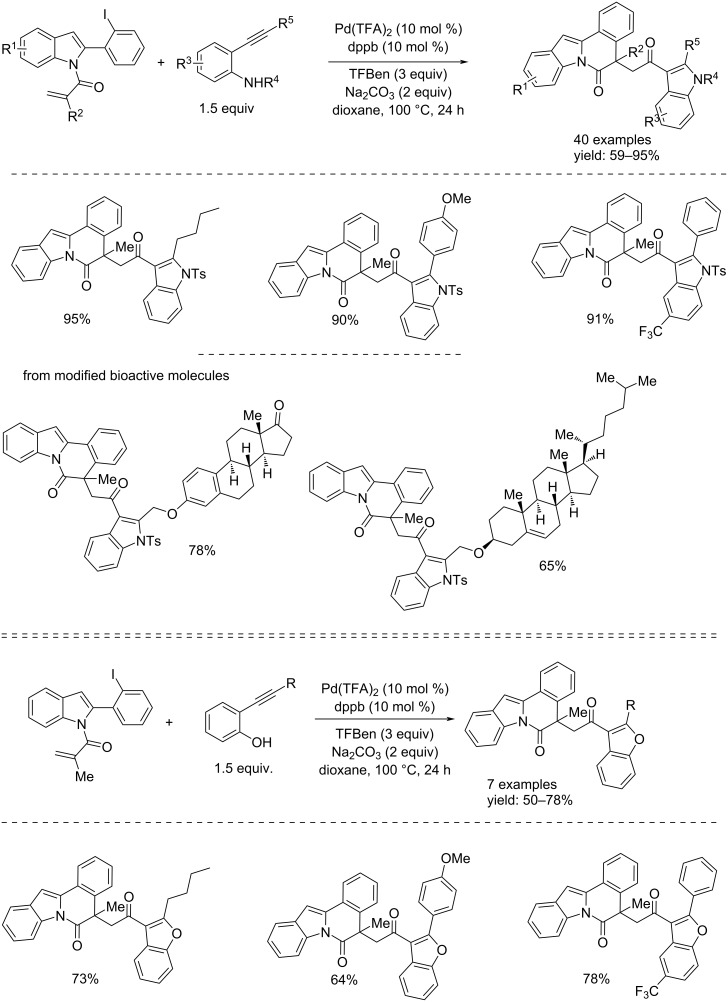
Pd(0)-catalyzed synthesis of CO-linked heterocyclic scaffolds from alkene-indole derivatives and 2-alkynylanilines (top) and 2-alkynylphenols (bottom).

**Scheme 34:**
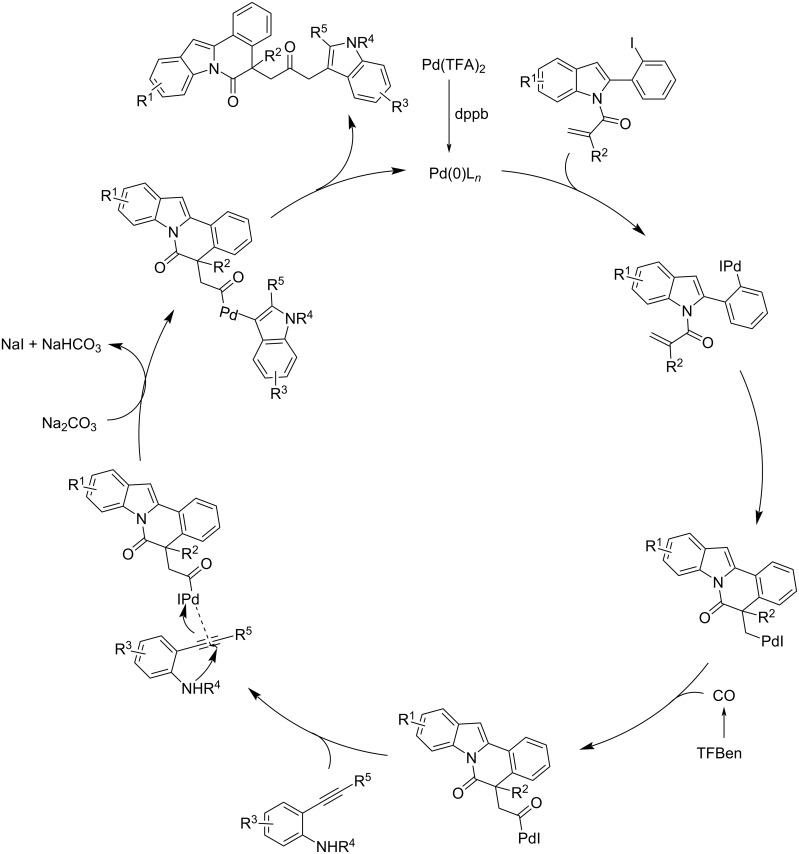
Proposed mechanism for the Pd(0)-catalyzed synthesis of CO-linked heterocyclic scaffolds.

About three years later, the same group developed a simple and efficient method to access CO-linked heterocyclic scaffolds by a Pd-catalyzed carbonylative cyclization of alkene–indole derivatives with 2-alkynylanilines and 2-alkynylphenols, in the presence of TFBen as CO source [63]. The reaction proceeded to heterocyclic compounds in the presence of 10 mol % of Pd(TFA)_2_ and dppb (1,4-bis(diphenylphosphino)butane), 3 equivalents of TFBen, 2 equivalents of Na_2_CO_3_ in dioxane at 100 °C. The products were obtained in very good isolated yields (Scheme 33). The proposed mechanism, shown in Scheme 34, suggested that the process proceeded through a Pd(0) catalysis proceeding through first an intramolecular Heck reaction, followed by CO insertion, *N*-cyclization (anilines) or *O*-cyclization (phenols) and final reductive elimination.

### Carbonylative functionalization of indoles to 3-substituted indoles

#### Functionalization through direct C–H alkoxycarbonylation

**Scheme 35:**
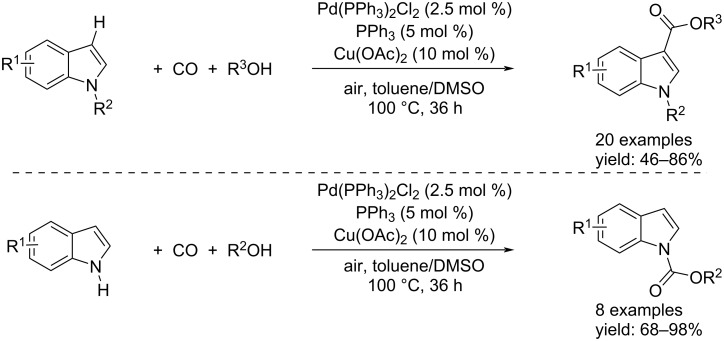
Pd-catalyzed C–H and N–H alkoxycarbonylation of indole derivatives to indole-3-carboxylates and indole-*N*-carboxylates reported by Lei et al. [66].

The transition-metal-catalyzed carbonylation of aryl halides, triflates, and tosylates with carbon monoxide and an alcohol was first pioneered by Heck and co-workers in 1974 [64–65]. Since then, this method has been well-developed and is considered one of the most straightforward ways to access carboxylic esters. In later years, other compounds were studied as substrates for alkoxycarbonylation processes. In fact, aromatic C–H functionalizations have been increasingly used for the synthesis of organic building blocks and pharmaceutical compounds. In this context, given the importance of indoles, in 2011, the groups by Lei and Li independently reported the C–H alkoxycarbonylation of indole derivatives [66–67]. In addition, Lei and co-workers also reported the N–H alkoxycarbonylation [66]. Both processes took place in a Schlenk tube loaded with 2.5 mol % of Pd(PPh_3_)_2_Cl_2_ as catalyst, 10 mol % of Cu(OAc)_2_ as co-catalyst, 5 mol % of PPh_3_ as ligand under a mixture of CO/air 7:1 in toluene/DMSO as solvent at 100 °C for 36 h. The desired products were isolated in yields up to 98% (Scheme 35).

**Scheme 36:**
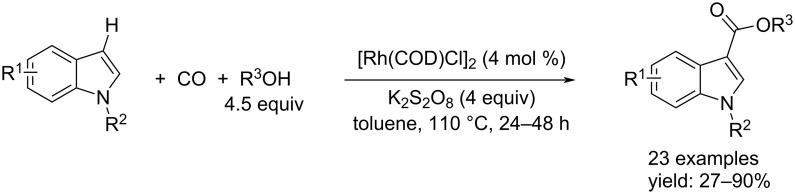
Rh-catalyzed C–H alcoxycarbonylation of indole derivatives to indole-3-carboxylates reported by Li et al. [67].

Unlike what we have just written, Li’s group performed the reaction by a Rh-catalysis using [Rh(COD)Cl]_2_ and re-oxidizing it with K_2_S_2_O_8_. The reactions led to the products in toluene after 24–48 hours at 110 °C under 1 bar of CO (Scheme 36).

**Scheme 37:**
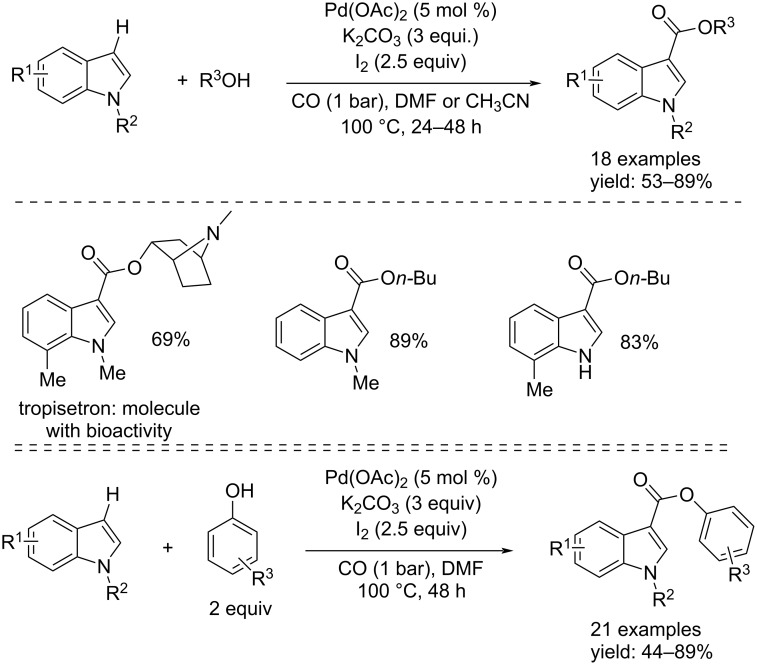
Pd-catalyzed C–H alkoxycarbonylation of indole derivatives with alcohols and phenols to indole-3-carboxylates.

Another example to obtain indole-3-carboxylates, was again reported by Li et al. [68]. The process occurred under mild conditions (such as the approaches seen above) under 1 bar of CO, in DMF or CH_3_CN, at 80–100 °C for 24–48 h. In addition to 5 mol % of Pd(OAc)_2_, 3 equiv of K_2_CO_3_ and 2.5 equiv of I_2_ were added. The reactions were carried out by using both aliphatic alcohols and phenols (Scheme 37) and good isolated yields were achieved.

**Scheme 38:**
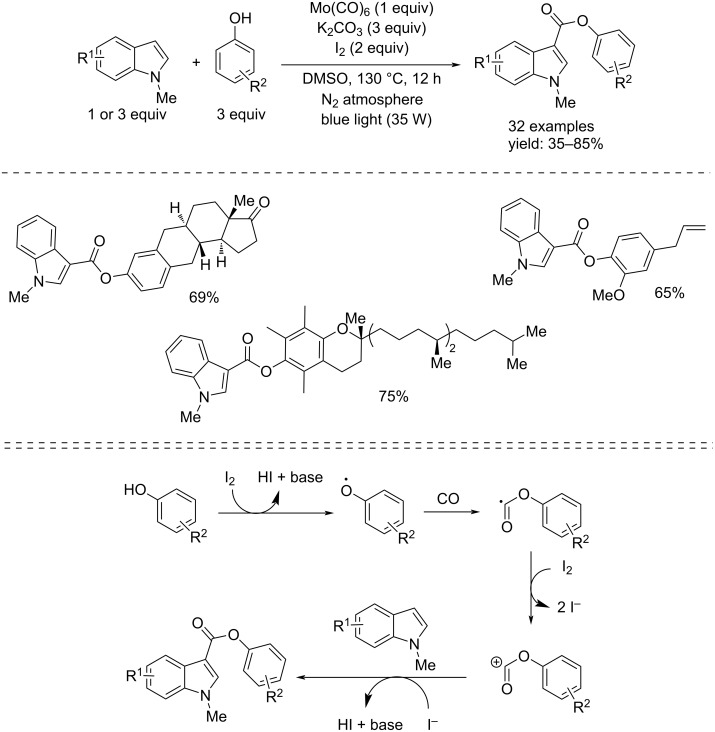
Synthesis of *N*-methylindole-3-carboxylates from *N*-methylindoles and phenols through metal-catalyst-free reaction (top) and its hypothesized mechanism (bottom).

In 2021, Peng and co-workers published a more environmentally friendly alkoxycarbonylation approach with phenols, from *N-*methylindoles, to synthesize *N*-methylndole-3-carboxylates without using of noble metal catalysts [69]. The process was catalyzed by visible light in the presence of Mo(CO)_6_ (1 equiv) as CO source, I_2_ (2 equiv), and K_2_CO_3_ (3 equiv) at 120 °C in an inert reaction environment (N_2_) and in DMSO as solvent (Scheme 38).

#### Functionalization through direct C–H carbonylations

The direct functionalization of indoles via C–C bond formation has received great interest due to the broad applications of functionalized indole derivatives. In this context, many groups developed different routes to 3-substituted indoles. In this mini-review, we just show the carbonylative approaches.

**Scheme 39:**
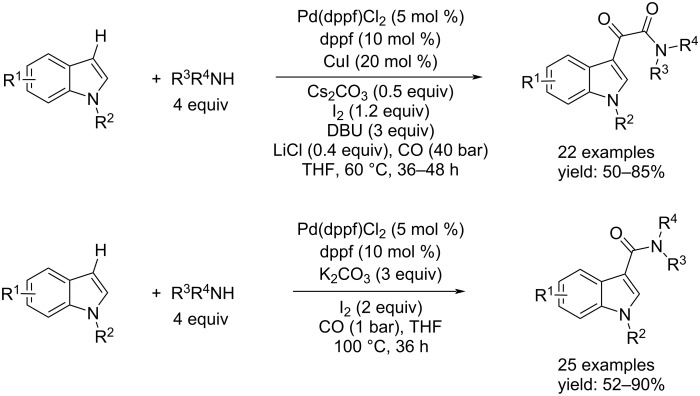
Synthesis of indol-3-α-ketoamides (top) and indol-3-amides (bottom) via direct double- and monoaminocarbonylation of indole derivatives with secondary amines.

Xing and co-workers presented a controllable and regioselective synthesis of indol-3-α-ketoamides and indol-3-amides via the direct double- and monoaminocarbonylation of indole derivatives by using secondary amines [70]. They used Pd(dppf)Cl_2_ as catalyst system. The indol-3-α-ketoamides were synthesized by adding dppf as ligand, CuI as co-catalyst, and LiCl as additive under 40 bar of CO. To make the process regioselective towards indol-3-amides, dppf as ligand, the base (K_2_CO_3_), and I_2_ were added, and the reaction run under a low pressure of CO (1 bar), in addition to the catalyst. Both processes took place well in THF (Scheme 39).

**Scheme 40:**
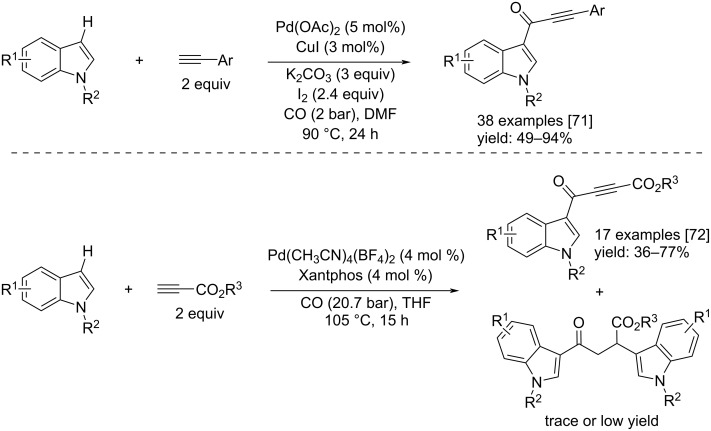
The direct Sonogashira carbonylation coupling reaction of indoles and alkynes via Pd/CuI catalysis reported by Li et al. [71] (top). The Pd-catalyzed regio- and chemoselective direct coupling of indoles/CO/alkynyl carboxylates developed by Zeng and Alper [72] (bottom).

In 2013, the Li’s group and Zeng and Alper developed two different methods for carrying out a direct carbonylation of indoles with alkynes. Li’s group reported the direct Sonogashira carbonylation coupling reaction of indoles and alkynes catalyzed by Pd/CuI in the presence of iodine as oxidant [71]. The catalyst system was Pd(OAc)_2_/CuI, in addition, a base (K_2_CO_3_) was added in DMF as solvent. Performing the reactions at 90 °C for 24 hours led to 38 products with isolated yields up to 94% (Scheme 40).

Instead, Zeng and Alper presented a new regioselective and chemoselective method for the direct coupling of indoles/CO/alkynes (alkynylcarboxylates) towards linear α,β-unsaturated ketones [72]. The reactions occurred in the presence of Pd(CH_3_CN)_4_(BF_4_)_2_/Xantphos as catalyst system under 20.7 bar of CO at 105 °C in THF. After 15 h, each reaction led selectively to the desired products (Scheme 40).

**Scheme 41:**
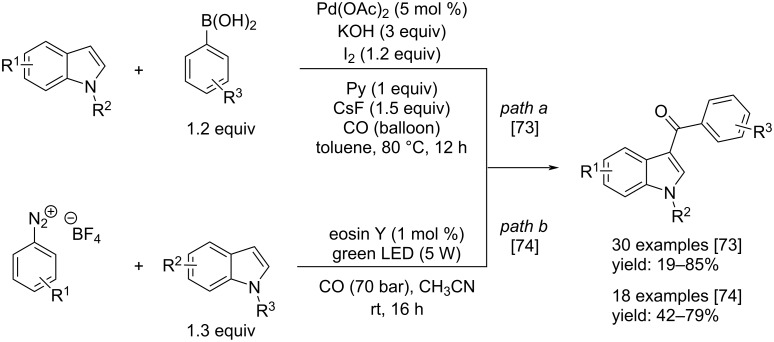
Synthesis of indole-3-yl aryl ketones reported by Zhao and co-workers [73] (path a) and Zhang and co-workers [74] (path b).

More recently, Zhao and co-workers published a novel synthesis of indole-3-yl aryl ketones by a Pd-catalyzed direct carbonylation of the corresponding indoles with boronic acids [73]. The best catalyst was Pd(OAc)_2_ which catalyzed the reaction well in the presence of a base, such as KOH, in toluene as solvent and by adding pyridine and CsF as additives. By this route the products were obtained under mild reaction conditions applying low pressure of CO, temperature of 80 °C for 12 h. A stoichiometric amount of I_2_ was necessary to restore the catalyst that underwent a possible reduction (Scheme 41).

Another interesting method toward indole-3-yl aryl ketones was reported by Zhang et al. Considering the ability of the aryldiazonium salts to act as aryl radical source, in presence of the suitable metal catalyst or taking advantage of photocatalysis, they decided to perform a direct carbonylation of indoles with that kind of chemicals [74]. The reaction was cheap because it took place by irradiation with green light (5 W) in the presence of eosin Y as photocatalyst, under 70 bar of CO, in CH_3_CN at room temperature (Scheme 41).

**Scheme 42:**
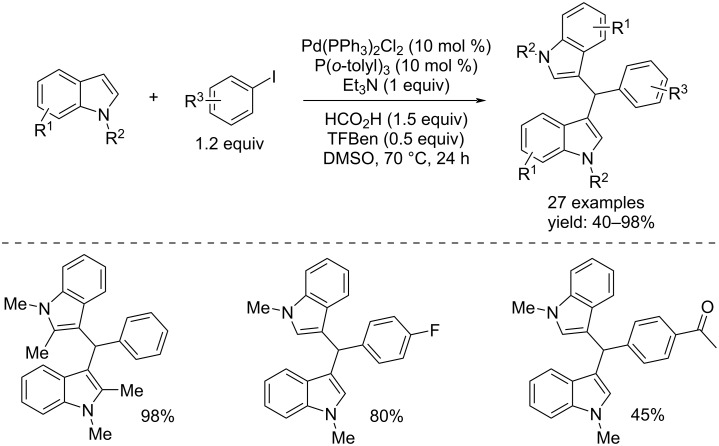
Pd-catalyzed carbonylative synthesis of BIMs from aryl iodides and *N*-substituted and NH-free indoles with of TFBen as CO source.

In 2018, Wu et al. published two papers about the functionalization of indoles. In the first one, based on the known bioactivity displayed by bis(indolyl)methane (BIM) compounds, they reported the Pd-catalyzed carbonylative synthesis of the target compounds from aryl iodides and *N*-substituted and NH-free indoles in the presence of TFBen as the CO source [75]. A wide range of bis(indolyl)methane compounds were isolated in moderate and excellent yields after 24 hours in DMSO, Et_3_N and formic acid as additives and, Pd(PPh_3_)_2_Cl_2_/P(*o*-tolyl)_3_ as catalyst system. The reaction conditions and some important BIMs synthesized are shown in Scheme 42.

**Scheme 43:**
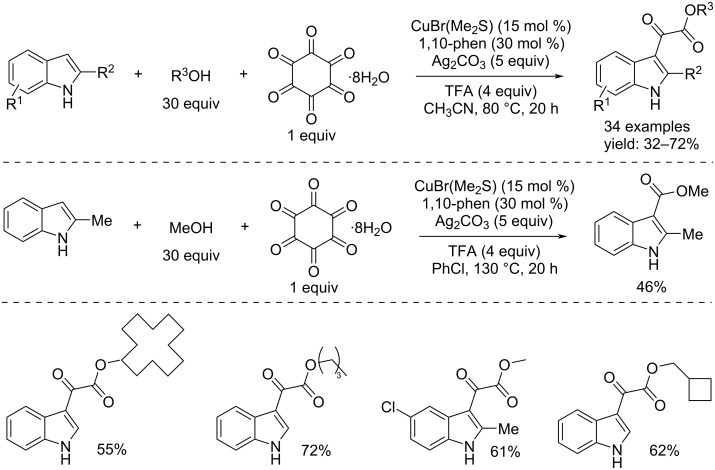
Cu-catalyzed direct double-carbonylation and monocarbonylation of indoles and alcohols with hexaketocyclohexane octahydrate as CO source.

In the second paper they used hexaketocyclohexane octahydrate as the CO source again. This cyclic hexaketone is a non-toxic stable solid and therefore, it is simple and safe to use unlike of carbon monoxide. It was used as reagent to obtain indol-α-ketoesters by the Cu-catalyzed direct double-carbonylation of indoles and alcohols [76]. The authors demonstrated that by adding Ag_2_CO_3_ in addition to CuBr(Me_2_S) as catalyst, 1-10-phen as ligand, and TFA as additive in CH_3_CN, the process led to the direct double-carbonylation products. Furthermore, they showed, through an example, that carrying out the reaction under the standard conditions but by changing the solvent (PhCl instead of CH_3_CN) and further increasing the temperature to 130 °C, the reaction took another pathway toward the direct monocarbonylation product, i.e. an indolester (Scheme 43).

### Carbonylative functionalization of indoles to 2-substituted indoles

As illustrated above, the metal-catalyzed direct C–H carbonylation of indoles is an effective and straightforward approach to synthesize indole derivatives containing a carbonyl function in position 3. On the other hand, only a few examples to synthesize indole-2-carbonyl derivatives have been realized.

One example is the regioselective Ru-catalyzed direct carbonylative arylation of heterocycles developed by Beller et al. in 2014 [77]. In this paper various heterocycles were investigated including five examples of indoles.

Another example was reported by Driver an co-workers, who developed the Pd-catalyzed direct aryl C–H aminocarbonylation with nitroarenes as nitrogen source [78]. In this case just one example of indole functionalization was reported.

**Scheme 44:**
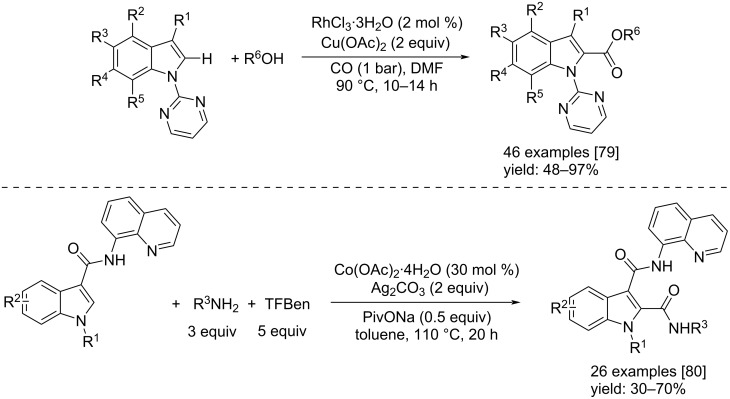
Rh-catalyzed direct C–H alkoxycarbonylation of indoles to indole-2-carboxylates [79] (top) and Co-catalyzed direct C–H aminocarbonylation of indoles to indole-2-amides [80] (below).

In 2019, Zhao and co-workers, reported an interesting method to synthesize indole-2-carboxylates through a Rh-catalyzed direct C–H alkoxycarbonylation of indoles [79]. The reaction took place under only 1 bar of CO and in the presence of RhCl_3_∙3H_2_O as catalyst and 2 equivalents of Cu(OAc)_2_ as oxidant. 44 examples were obtained in good to excellent isolated yields (Scheme 44). Nevertheless, the approaches by Zhao et al. and the other examples, have in common the use of expensive catalysts. Therefore, Wu’s group developed the direct C–H aminocarbonylation of indoles by using cheaper Co(OAc)_2_·4H_2_O as catalyst [80] which allowed the reaction to proceed well when used in conjunction of Ag_2_CO_3_ as oxidant and a further addition of PivONa as additive. The presence of CO as reagent was guaranteed by the addition of TFBen. The reaction conditions are reported in Scheme 44.

### Carbonylative functionalization of indoles toward 4 to 7-substituted indoles

**Scheme 45:**
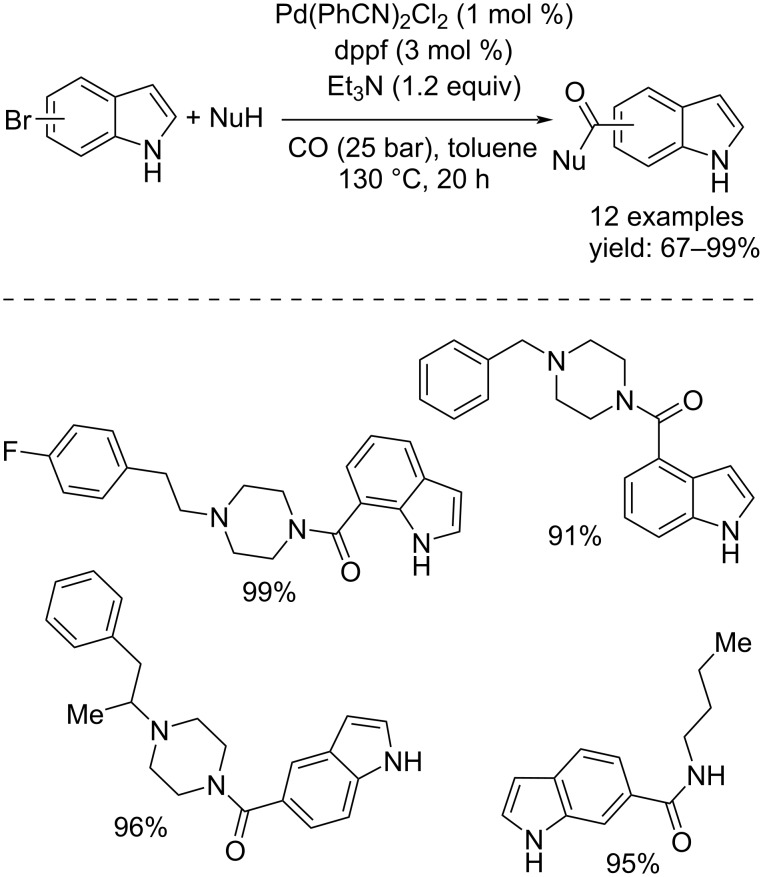
Pd-catalyzed carbonylation of NH free-haloindoles.

In 2004, Beller and co-workers evaluated the possibility of synthesizing indole carboxylic amides from 4 to 7-haloindoles through a Pd-catalyzed carbonylation reaction [81]. Initially it was assumed that the presence of the halide and the NH group could lead to an oligomerization or polymerization reaction, but carrying out the reaction with Pd(CH_3_CN)_2_Cl_2_/dppf as catalyst system and Et_3_N as the base at 130 °C under 25 bar of CO, they succeeded in the synthesis of a set of 12 desired products. These included an ethyl ester and a carboxylic acid, and were all obtained in good yields of up to 99% (Scheme 45).

## Conclusion

We have summarized the importance of carbon monoxide as C1 building block to promote different kinds of transformations to synthesize and functionalize indole scaffolds. We have seen different approaches in which high catalyst efficiencies, mild conditions and the use of low-toxic chemicals as CO source, that do not require the use of autoclaves, have been successfully applied. Furthermore, in some cases, “green” oxidants such as air were used making the process more environmentally friendly especially if we think about them in the industrial field. Clearly, the research in this field is ongoing and it will continue, and more active catalysts and new “green” synthetic approaches will be developed.

## Data Availability

Data sharing is not applicable as no new data was generated or analyzed in this study.
